# Artificial intelligence-based Raynaud’s quantification index (ARTIX): an objective mobile-based tool for patient-centered assessment of Raynaud’s phenomenon

**DOI:** 10.1186/s13075-025-03569-w

**Published:** 2025-06-03

**Authors:** Marco Di Battista, Seda Colak, Anna Howard, Francesca Donadoni, Chris Owen-Smith, Andrea Rindone, Stefano Di Donato, Collette Hartley, Lesley-Anne Bissell, Francesco Del Galdo

**Affiliations:** 1 Leeds Institute of Rheumatic and Musculoskeletal Medicine, University of Leeds, Leeds, LS7 4SA UK; 2 Rheumatology Unit, University of Pisa, Pisa, Italy; 3 Department of Rheumatology, Gulhane Training and Research Hospital, Ankara, Turkey; 4 NIHR Leeds Biomedical Research Centre, Leeds Teaching Hospitals Trust, Leeds, UK; 5 Procedure Health Limited, London, UK; 6 Department of Rheumatology and Medical Science, University of Milan, ASST Gaetano Pini- CTO Institute, Milan, Italy

**Keywords:** Raynaud’s phenomenon, Systemic sclerosis, Artificial intelligence, Thermography, Cold challenge

## Abstract

**Background:**

We aimed to develop an artificial intelligence algorithm able to assess Raynaud’s phenomenon (RP) from mobile phone photography, ensuring as a patient-centered, image-based method for RP quantification.

**Methods:**

ARTIX (artificial intelligence-based Raynaud’s quantification index) score was developed as a multi-step process of segmentation, decomposition and filters application to mobile phone pictures of the hand. ARTIX was validated by the ability to assess finger response to standardised cold challenge in patients with primary and secondary RP and healthy controls (HC) and compared with thermography as a reference.

**Results:**

Forty-five RP patients (91.1% female, mean age 52.2 years, 75.5% secondary RP) were enrolled, along with 22 HC comparable for age and gender. RP patients presented significantly lower ARTIX values than HC both at baseline (*p* < 0.001) and across all timepoints of the cold challenge (*p* < 0.01 for all), paralleling a similarly significant difference observed by thermography. ARTIX score was higher in males and in patients taking vasoactive drugs, whereas lower values were obtained in patients with late capillaroscopic pattern, diffuse cutaneous skin subset, or negative for anti-centromere antibodies. ARTIX showed also good ability to discriminate between RP and HC response to cold challenge.

**Conclusion:**

We developed and validated ARTIX, a novel machine learning-driven method for the objective quantification of RP. Real-life longitudinal studies in patients with RP will determine the value of ARTIX to complement patient self-assessment surrogate measures of RP activity and severity.

**Supplementary Information:**

The online version contains supplementary material available at 10.1186/s13075-025-03569-w.

## Background

Raynaud’s phenomenon (RP) is a transient vasospastic disorder in which reduced blood flow to the extremities occurs. It is classified into primary and secondary RP depending on the presence of an underlying disease [1]. RP is among the clinical hallmarks of systemic sclerosis (SSc), and it is one of the red flags needed for the diagnosis of very early forms of the disease (VEDOSS) [2, 3]. RP can manifest with different degrees of severity, determining a potential burden on function, work, social participation and quality of life in patients [4]. Given all these implications and its recurrent nature, patient-centered assessment of RP severity is a crucial issue. So far, several patient-reported outcomes for the self-monitoring of RP have been proposed [5, 6], but objective tools to aid in the quantification of overall disease burden are currently lacking.

There is growing interest in developing algorithms that can aid clinical practice, particularly making use of artificial intelligence (AI). This has rapidly gained an important role in academic research, while also opening a new field of commercial applications. The ability of machine learning in dissecting patterns within a dataset has supported its employment in several aspects of data-related applications in medicine and healthcare, including the interpretation and quantification of visual data [7]. Independently of machine learning and preceding its deployment, the global uptake of mobile phone technology has been boosting the ability of devolved data collection, empowering patients both in “feeding” and controlling their own health data [8]. In recent years, patients with RP have increasingly utilized mobile technology to document their episodes collecting image proofs of their attacks, providing clinicians with visual evidence that can aid in diagnosis. These images capture the characteristic colour changes associated with RP, which reflect underlying alterations in blood flow and vascular reactivity, thus informing the rationale for this study. Given the potential of these images to serve as an objective marker of RP severity, we sought to develop a machine learning-driven algorithm capable of detecting and quantifying RP using mobile phone photography. This tool is designed to inform a patient-centered approach by enabling an image-based, objective quantification of RP burden.

## Methods

### ARTIX development

The study utilized a multi-step computational pipeline to analyse hand images for finger segmentation and color intensity analysis. This pipeline consisted of several key stages: hand segmentation using a UNet-based model, hand landmark detection with the MediaPipe framework, finger segmentation, and colour analysis of the segmented finger regions.

**Fig. 1:**
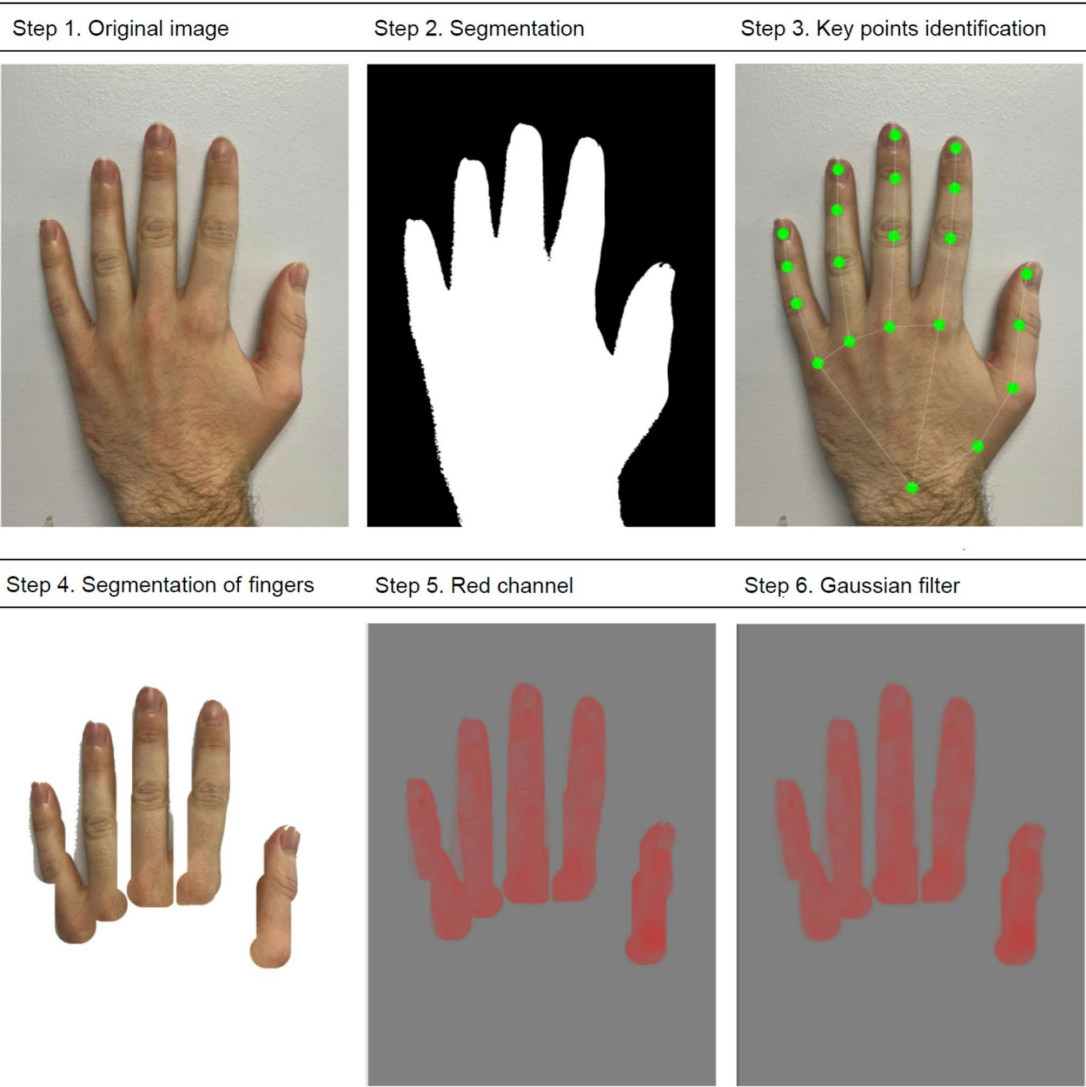
Preliminary steps for ARTIX development

All computational tasks were implemented using Python version 3.7 (*The Python Software Foundation*,* Wilmington*,* DE*,* USA*). The pipeline leveraged state-of-the-art machine learning libraries, including PyTorch for model training and evaluation, and MediaPipe for real-time landmark detection [9]. We employed a UNet-based model to segment hand regions from complex image backgrounds [10]. The UNet architecture was fine-tuned on mobile phone pictures of hands from healthy subjects already stored in our developer cloud. When evaluated on a separate validation set that was not used during training, the model demonstrated a high level of accuracy in identifying hand regions, with an overlap exceeding 95% between the predicted and actual hand areas. The model was implemented using the PyTorch library in Python and utilized a DeepLabV3 ResNet-50 backbone. Input images were first resized to a square dimension to ensure uniform processing. The model output binary masks delineating hand regions, which served as input for subsequent steps. To enhance segmentation precision, we employed the MediaPipe framework for real-time hand tracking and landmark detection. The MediaPipe Hand Landmarker module was utilized to identify key anatomical landmarks on the hand, including fingertips and joints. These landmarks provided crucial reference points for accurate finger segmentation. Finger segmentation was accomplished by leveraging the hand landmarks detected in the previous step. The landmarks were rescaled and aligned with the segmented hand masks to delineate individual finger regions. Custom algorithms were developed to extract masks for each finger on both the left and right hands, ensuring that each finger was accurately segmented from the hand. Once finger regions were segmented, colour channel statistics were computed for each finger region. The RGB colour channels (red, green, and blue) were extracted separately, and statistical measures such as the median and quartiles values were calculated. These statistics were used to characterize the distribution of colour intensities within each finger region. The analysis included a Gaussian filtering step to enhance robustness by mitigating the impact of noise and outliers. This filtering operation effectively removed outliers and noise, ensuring more accurate quantification of colour statistics and facilitating reliable analysis of colour distribution patterns. To quantify finger redness, which was prioritized as the most informative by the machine learning approach, the red colour channel was processed to compute redness metrics. A custom redness estimation algorithm was developed, which included smoothing the red channel data and calculating a redness score based on the median and quartile values (Fig. 1). The green and blue channels were also analyzed to characterize the distribution of colour intensities in the finger regions, thus improving the robustness of the algorithm.

Features captured in each segmented finger region were averaged in a single score that we named ARTIX (artificial intelligence-based Raynaud’s quantification index– property of University of Leeds, UK and Procedure Health Limited, London, UK). The ARTIX score, expressed in arbitrary units, encapsulates the colour intensity distribution within each finger region, excluding the thumbs, providing a single metric for quantitative comparisons across different images and patient groups.

Once developed, ARTIX was validated against multiple ground truths. As a clinical truth, we evaluated ARTIX performance in the comparison between RP patients and healthy controls (HC). Cold challenge and thermography were chosen as procedural and reference evaluative truths given their well-known validity and reliability in the assessment of Raynaud’s [11, 12].

### Study population

Adult consecutive patients with RP attending the Raynaud’s and Scleroderma Clinic of the University of Leeds were enrolled, along with a group of HC comparable for age and gender. The classification between primary RP and secondary RP was made according to regular clinical practice on clinical, laboratory and capillaroscopic evaluations carried out by the team of rheumatologists who follow the patients in the clinic. The enrolment took place in two different seasonal periods of 2023, from May to July and from September to December. This study was conducted as part of thermography analysis in the observational study STRIKE (IRAS ID: 178638; REC Reference: 15/NE/0211).

Epidemiological data, autoantibody profile, nailfold videocapillaroscopy (NVC) pattern according to Cutolo [13] and current vasoactive treatment were extracted by STRIKE database for analysis. NVC had been performed as per routine clinical practice by a dedicated rheumatologist using a videocapillaroscope at 200x magnification analysing the II to V fingers bilaterally. Subjects were further classified according to fulfilment of 2013 ACR/EULAR classification criteria or VEDOSS criteria [14, 15]. None of them discontinued their ongoing vasoactive therapy. Patients with SSc were annotated for cutaneous subset (limited - lcSSc or diffuse - dcSSc) and history of digital ulcers (DUs). Patients with fingers locked in flexion for contractures were excluded from the analysis.

### Cold challenge

Cold challenge was performed as previously standardised [9]. Briefly, subjects were asked not to smoke or consume caffeine or alcohol for 3 h prior to the examination. After 10 min of acclimatization, patient’s hands were first placed on a black, thermally insulated surface for the basal assessment. The dorsal side of both hands was then imaged with a mobile thermal camera (*FLIR C3*,* FLIR Systems*,* Kings Hill*,* UK*) and a mobile phone taking photographs with a resolution of 3000 × 4000 pixels (*iPhone 8*,* Apple*,* Cupertino*,* CA*,* USA* or *Redmi Note 7*,* Xiaomi*,* Beijing*,* China*). The thermal camera was quality-assured against a blackbody source prior to use.

Patients were then asked to wear nitrile gloves. Both hands were submerged to the metacarpophalangeal joints into cooled water at 15 ± 1 °C for 1 min. The gloves were then removed and the hands were returned to their original position on the insulating surface, performing a new assessment with both cameras right after the cold challenge and then every 2 min up to 10 min, thus obtaining a total of seven assessments for each patient.

### Thermography

Thermal images were analysed with a dedicated software (*FLIR Thermal Studio*,* Teledyne FLIR*,* Kings Hill*,* UK*) and regions of interest were drawn from the second to fifth finger bilaterally from the metacarpophalangeal joint to the fingertip, hence obtaining the mean temperature (°C) for each finger. Finally, the mean of the thermal values of the 8 fingers was calculated, thus obtaining a single thermal value per patient per each timepoint.

### Statistical analysis

Continuous data were described by mean and standard deviation, categorical data by absolute and relative frequency. The distribution of continuous variables was assessed by inspection of quantile–quantile plots; in doubtful cases Shapiro-Wilk’s test was used. Given the lack of similar prior studies, we have based our sample size on a rule of thumb for pilot studies of total *N* ≥ 55 as suggested by Sim and Lewis [16]. Pearson’s or Spearman’s correlation coefficients were computed to assess relationship between continuous variables as appropriate, according to data distribution and presence of outliers. One-way ANOVA with Bonferroni post-hoc correction and Student’s t-test for independent samples (two-tailed) were used to compare continuous data between groups when normally distributed. Kruskal-Wallis test with post-hoc Dunn’s test and Mann-Whitney U test for independent samples (two-tailed) were instead used for non-normally distributed continuous data. Chi square test of independence and McNemar’s test were used to analyse independent and paired categorical data, respectively. After excluding problems of collinearity calculating the variance inflation factor, a multilinear regression was performed to evaluate the influence on the ARTIX score of clinical variables with significance at univariate analysis and epidemiological factors. ARTIX classification accuracy was evaluated with ROC analysis and confusion matrix, reporting for each timepoint the Area Under Curve (AUC) with its 95% confidence interval and the percentages of sensitivity and specificity. Significance was set at 0.05 and all analyses were carried out with R software (*R Core Team 2023*,* R Foundation for Statistical Computing*,* Vienna*,* Austria*).

## Results

### Study population

Forty-five RP patients (91.1% female, mean age 52.2 ± 16.7 years) were enrolled, along with 22 HC comparable for age and gender. Table 1 summarizes clinical characteristics of the cohort. Briefly, three quarters of patients were classified as having VEDOSS (*n* = 15) or SSc (*n* = 19), with a high prevalence for anti-centromere autoantibodies (ACA– 42.2%). Among SSc patients, the most common skin subset was the limited one (*n* = 14) and 7 subjects had previously presented DUs. More than a half of the entire RP population was taking vasoactive drugs, mainly calcium channel blockers (33.3%) and phosphodiesterase-5 inhibitors (22.2%). The percentage of subjects taking beta-blockers, antiplatelets or anticoagulants was comparable between the two RP and HC subgroups, and less than 5% in both.

**Table 1:** Epidemiological and clinical characteristics of the cohort

	RP (*n* = 45)	HC (*n* = 22)	*p*
Female, n (%)	41 (91.1)	19 (86.4)	0.7
Age, mean (SD), years	52.2 (16.7)	45.7 (13.5)	0.1
Primary RP, n (%)	11 (24.5)		
VEDOSS, n (%)	15 (33.3)		
Systemic sclerosis, n (%)	19 (42.2)		
- limited cutaneous SSc, n (%)	14 (31.1)		
- diffuse cutaneous SSc, n (%)	5 (11.1)		
- history of digital ulcers, n (%)	7 (15.5)		
Autoantibody profile			
- anti-centromere, n (%)	19 (42.2)		
- anti-topoisomerase I, n (%)	5 (11.1)		
- anti-RNA polymerase III, n (%)	2 (4.4)		
- anti-PM/Scl, n (%)	2 (4.4)		
Capillaroscopy pattern (some missing)			
- early, n (%)	6 (13.3)		
- active, n (%)	3 (6.6)		
- late, n (%)	5 (11.1)		
- non-specific, n (%)	7 (15.5)		
Ongoing vasoactive therapy, n (%)	25 (55.5)		
- calcium channel blockers, n (%)	15 (33.3)		
- endothelin receptor antagonists, n (%)	2 (4.4)		
- phosphodiesterase-5 inhibitors, n (%)	10 (22.2)		
- iloprost, n (%)	3 (6.6)		

RP: Raynaud’s phenomenon; HC: healthy controls; VEDOSS: very early disease of systemic sclerosis; SSc: systemic sclerosis

### ARTIX vs. thermography

**Fig. 2:**
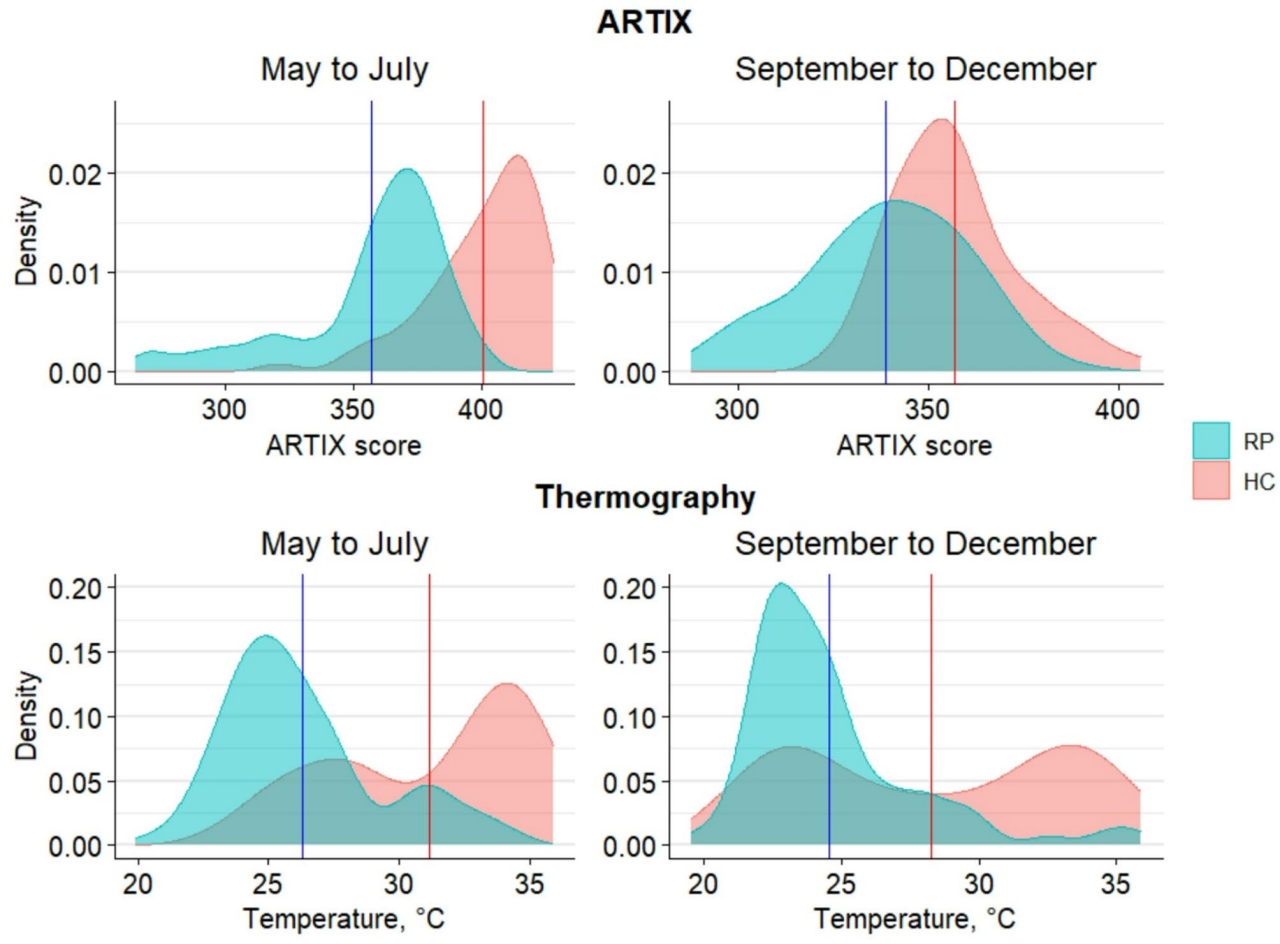
Density plots showing comprehensive differences (mean highlighted) between RP and HC results both for ARTIX and thermography regardless of seasonal variability

At baseline, RP showed significantly lower values than HC both at ARTIX (344 ± 29 vs. 373 ± 33; *p* = 0.001) and thermography (27.9 ± 2.5 °C vs. 30.8 ± 3.0 °C; *p* < 0.001). At a comprehensive evaluation of all timepoints, RP presented lower ARTIX values than HC (348 ± 28 vs. 377 ± 29; *p* < 0.001). This was paralleled by thermography (25.5 ± 3.2 °C vs. 29.6 ± 4.6 °C; *p* < 0.001). Seasonal influence was found to be widely significant, keeping the same pattern for both methods. In fact, subjects evaluated from May to July (*n* = 33) presented higher values than those assessed from September to December (*n* = 34) both for ARTIX (370 ± 35 vs. 345 ± 22; *p* < 0.001) and thermography (27.8 ± 4.0 °C vs. 25.9 ± 4.2 °C; *p* < 0.001). These results were confirmed when considering only patients with RP or HC (*p* < 0.001 for all). Specifically, the significant difference between RP and HC, with the former having lower values, was maintained despite seasonal variability in both methods as shown in Fig. 2.

No correlation with age was found for either technique (*r* = 0.07 with *p* = 0.1 for ARTIX and *r*= -0.03 with *p* = 0.5 for thermography). Overall, female subjects showed significantly lower values than males both at ARTIX (356 ± 32 vs. 370 ± 23; *p* < 0.001) and thermography (26.5 ± 4.1 °C vs. 29.6 ± 3.6 °C; *p* < 0.001). These results were confirmed when considering only patients with RP (347 ± 28 vs. 359 ± 21 with *p* = 0.008 for ARTIX, and 25.1 ± 3.0 °C vs. 28.6 ± 3.2 °C with *p* < 0.001 for thermography), whereas the significance was lost for both methods in HC (376 ± 30 vs. 383 ± 19 with *p* = 0.1 for ARTIX, and 29.3 ± 4.7 °C vs. 31.0 ± 3.7 °C with *p* = 0.1 for thermography).

**Fig. 3:**
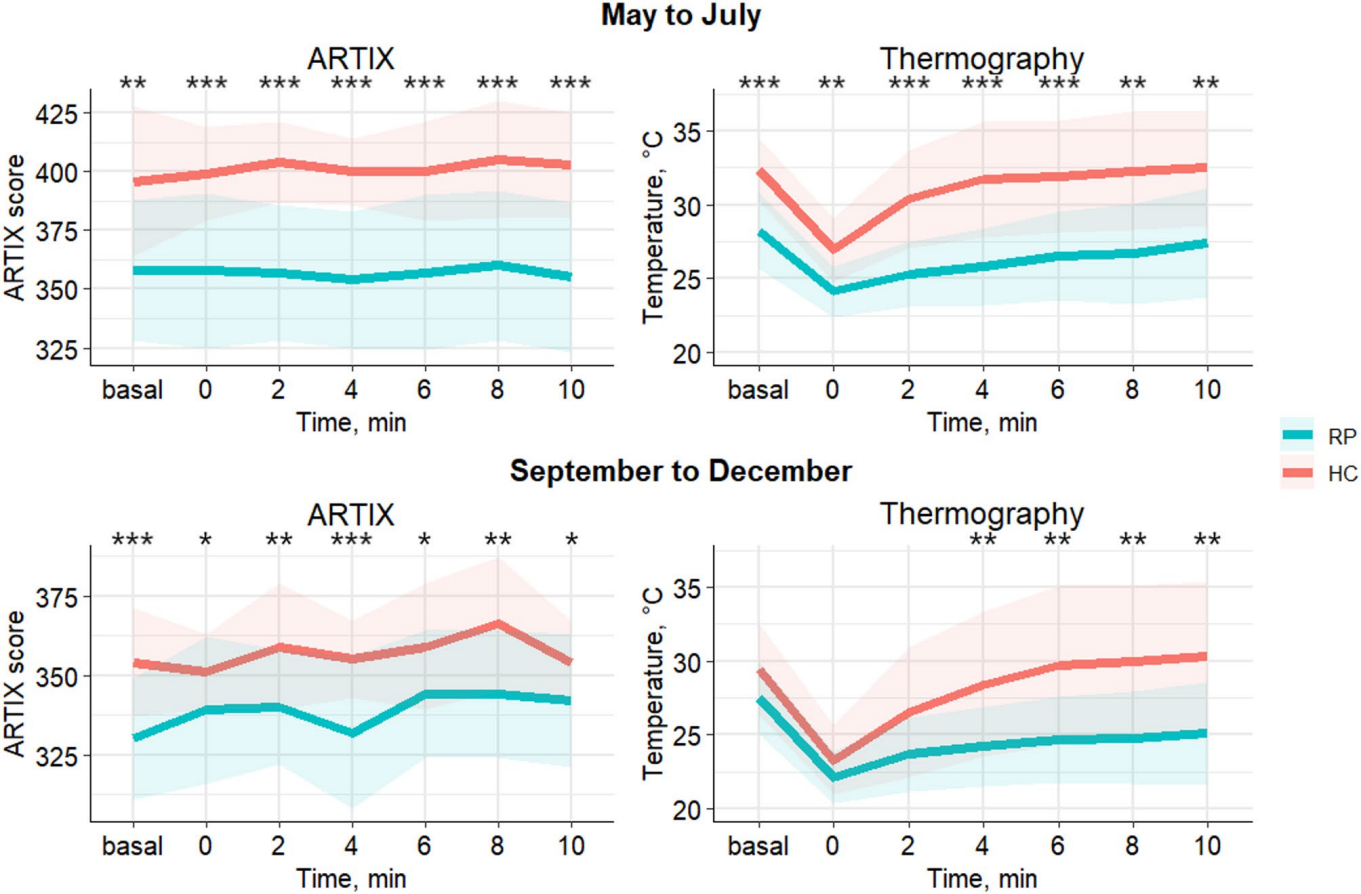
Mean trends during cold challenge for ARTIX and thermography showing constant differences between RP and HC regardless of seasonal variability. * *p* ≤ 0.05; ** *p* ≤ 0.01; *** *p* ≤ 0.001

Comparisons were then evaluated for each timepoint of the cold challenge. As reported in Table 2, RP showed constantly lower ARTIX scores than HC (*p* < 0.01 throughout the test). These ARTIX results were confirmed significant for each timepoint even when differentiating for seasonal variability. As shown in Fig. 3, ARTIX difference between RP and HC paralleled the outcomes observed with thermography both in the period from May to July and from September to December. Of note, thermography failed to detect a difference between RP and HC at the first three timepoints of the cold challenge performed from September to December, whereas ARTIX showed significance there as well (Table 2).

**Table 2:** Comparisons of ARTIX and thermography results between RP and HC across the timepoints of cold challenge, highlighting global outcomes and seasonal differences

ARTIX									
*Time*	**Global**			**May to July**			**September to December**		
	*RP* *(* *n* *= 45)*	*HC* *(* *n* *= 22)*	*p*	*RP* *(* *n* *= 23)*	*HC* *(* *n* *= 10)*	*p*	*RP* *(* *n* *= 22)*	*HC* *(* *n* *= 12)*	*p*
Basal	344 (29)	373 (33)	**0.001**	358 (30)	396 (32)	**0.006**	330 (19)	354 (17)	**0.001**
0 min	349 (30)	373 (29)	**0.003**	358 (33)	399 (20)	**< 0.001**	339 (23)	351 (12)	**0.04**
2 min	349 (25)	380 (30)	**< 0.001**	357 (29)	404 (17)	**< 0.001**	340 (18)	359 (20)	**0.01**
4 min	344 (29)	375 (27)	**< 0.001**	354 (29)	400 (14)	**< 0.001**	332 (24)	355 (12)	**< 0.001**
6 min	351 (28)	378 (29)	**< 0.001**	357 (33)	400 (21)	**< 0.001**	344 (20)	359 (20)	**0.03**
8 min	352 (28)	384 (30)	**< 0.001**	360 (32)	405 (25)	**< 0.001**	344 (20)	366 (21)	**0.008**
10 min	349 (28)	376 (30)	**< 0.001**	355 (32)	403 (22)	**< 0.001**	342 (21)	354 (13)	**0.05**
**Thermography**									
Basal	27.9 (2.5)	30.8 (3.0)	**< 0.001**	28.2 (2.6)	32.4 (2.1)	**< 0.001**	27.5 (2.4)	29.4 (3.1)	n.s.
0 min	23.1 (2.0)	25.0 (2.9)	**0.01**	24.1 (1.7)	27.0 (2.2)	**0.002**	22.1 (1.8)	23.3 (2.3)	n.s.
2 min	24.5 (2.5)	28.3 (4.3)	**< 0.001**	25.3 (2.2)	30.4 (3.3)	**< 0.001**	23.7 (2.5)	26.5 (4.4)	n.s.
4 min	25.0 (2.8)	29.9 (4.7)	**< 0.001**	25.8 (2.6)	31.7 (3.9)	**< 0.001**	24.2 (2.7)	28.4 (4.9)	**0.01**
6 min	25.6 (3.1)	30.7 (4.8)	**< 0.001**	26.5 (3.0)	31.9 (3.8)	**0.001**	24.7 (2.9)	29.7 (5.4)	**0.009**
8 min	25.8 (3.4)	31.0 (4.6)	**< 0.001**	26.7 (3.4)	32.3 (4.0)	**0.002**	24.8 (3.1)	30.0 (5.1)	**0.005**
10 min	26.3 (3.7)	31.3 (4.6)	**< 0.001**	27.4 (3.7)	32.5 (3.9)	**0.003**	25.1 (3.5)	30.3 (5.0)	**0.005**

Data are reported in mean (SD)

**Fig. 4:**
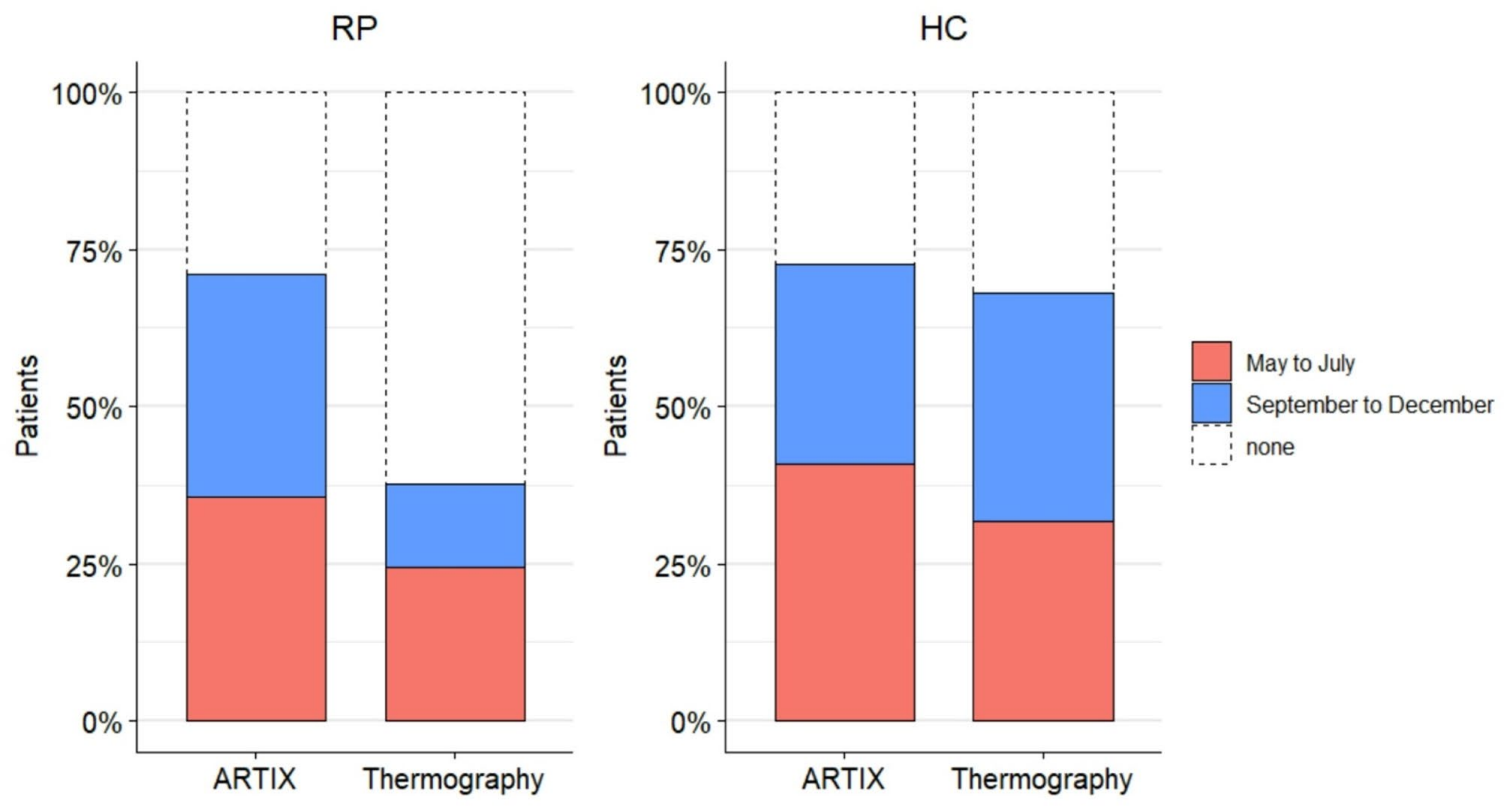
Differences between ARTIX and thermography in the proportions of subjects who returned to baseline values at the 10th minute of cold challenge

The percentage of patients who returned to (including those who eventually have gone beyond) baseline values at the tenth minute of cold challenge was assessed. As reported in Table 3, 71.1% of all RP returned to baseline ARTIX values, whereas only 37.8% patients returned to baseline temperatures (*p* = 0.003). In contrast, the percentages of HC returning to baseline values at the tenth minute were similar between ARTIX and thermography (72.7% vs. 68.2%; *p* > 0.9). When differentiating for seasonal periods, a significance was solely observed in the proportion of RP returning to baseline ARTIX values from September to December compared to thermography (*p* = 0.01). Conversely, thermography showed that fewer RP returned to baseline values than HC both globally and from September to December (*p* = 0.02 and *p* = 0.03, respectively), whereas ARTIX did not show any significant difference (Fig. 4).

**Table 3:** Proportions of subjects who returned to baseline values at the 10th minute of cold challenge

		ARTIX	Thermography	*p*
Global	RP (*n* = 45)	32 (71.1%)	17 (37.8%)	**0.003**
	HC (*n* = 22)	16 (72.7%)	15 (68.2%)	n.s.
	**p**	n.s.	**0.02**	
May to July	RP (*n* = 23)	16 (69.6%)	11 (47.8%)	n.s.
	HC (*n* = 10)	9 (90%)	7 (70%)	n.s.
	**p**	n.s.	n.s.	
September to December	RP (*n* = 22)	16 (72.7%)	6 (27.3%)	**0.01**
	HC (*n* = 12)	7 (58.3%)	8 (66.7%)	n.s.
	**p**	n.s.	**0.03**	

### RP-specific characteristics

When assessing ARTIX relationship with RP-specific characteristics, we did not observe any statistical difference between primary and secondary RP (342 ± 32 vs. 350 ± 26; *p* = 0.06). On the other side, RP patients who were taking vasoactive therapy exhibited greater overall ARTIX values (352 ± 24 vs. 343 ± 32; *p* = 0.003).

When considering the subgroup of secondary RP (VEDOSS + SSc, *n* = 34), ACA positivity was associated with significantly higher ARTIX results than other autoantibodies (353 ± 25 vs. 338 ± 29; *p* < 0.001). Moreover, NVC showed that patients with more advanced microvascular damage (late scleroderma pattern) had significantly lower ARTIX values than those in the earlier stages (scleroderma pattern: early or active) (353 ± 15 vs. 365 ± 27; *p* = 0.03).

**Fig. 5:**
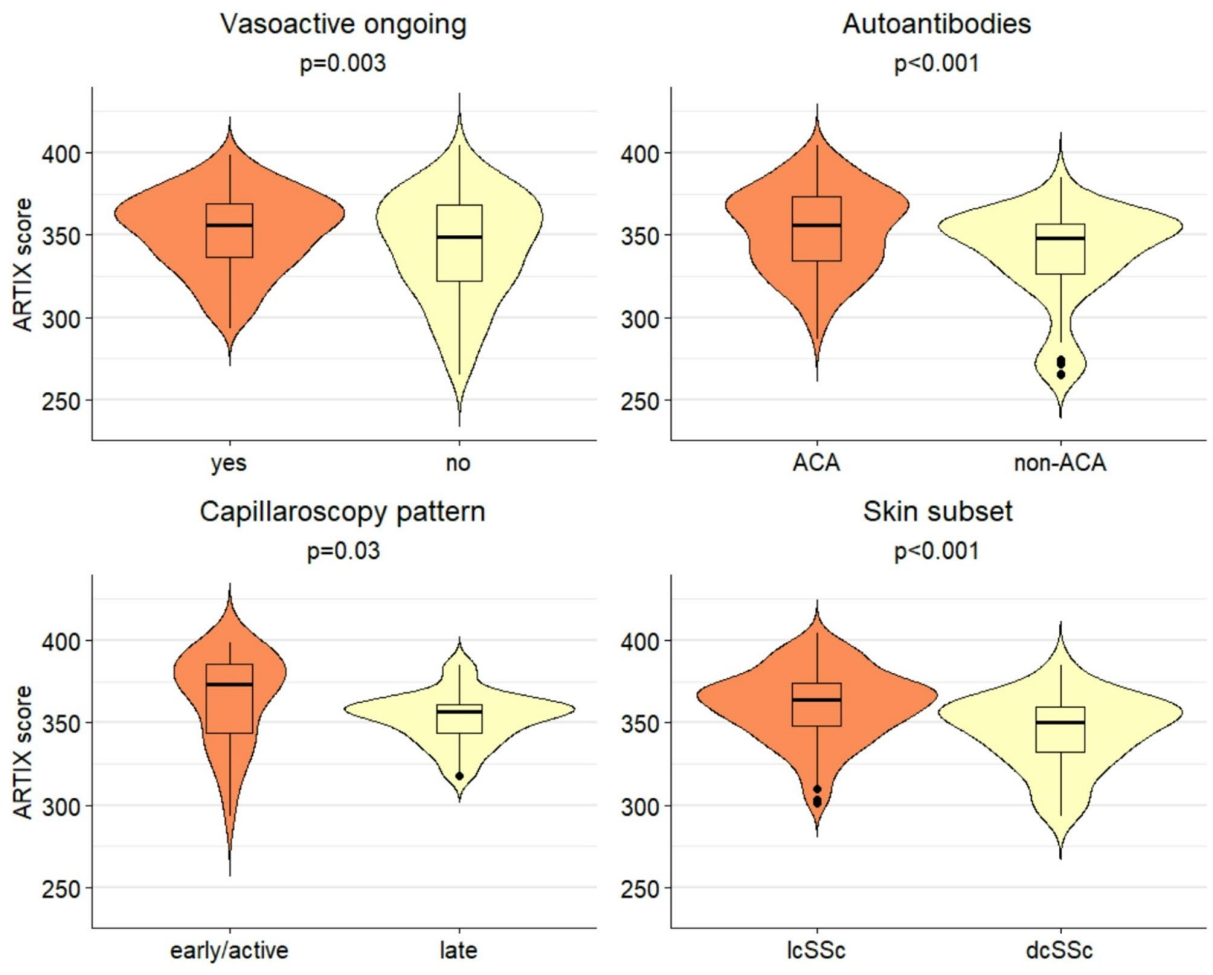
Significant clinical associations for ARTIX score in patients with secondary RP (summary statistics shown by box plots and data distribution by violin plots)

In the subgroup of patients with SSc, the diffuse cutaneous subset was associated with lower ARTIX values (345 ± 22 for dcSSc vs. 360 ± 22 for lcSSc; *p* < 0.001). No significant differences were found regarding the history of DUs (354 ± 24 for presence vs. 357 ± 22 for absence; *p* = 0.4). Figure 5 depicts all the significant clinical associations with ARTIX score.

A multivariate analysis was then performed taking into account ongoing vasoactive therapy, ACA positivity, NVC pattern and skin subset, along with age and gender. The multilinear regression confirmed the significant influence of ongoing vasoactive treatment (B = 13.8; *p* = 0.003) and ACA positivity (B = 19.9; *p* < 0.001) on ARTIX.

### ARTIX classification accuracy

Finally, we investigated ARTIX accuracy to discriminate between RP and HC. As reported in Table 4, ARTIX showed a good performance at ROC analysis across all the timepoints of the cold challenge, with an AUC almost always higher than 0.7. This translated into a very high sensitivity greater than 90% for each timepoint, whereas the specificity ranged from 36.4 to 54.6%. The best timepoint for ARTIX accuracy was 2 min after the cold challenge with AUC 0.773 (95%CI 0.643–0.904), sensitivity 95.6% and specificity 54.6%.

**Table 4:** ARTIX classification accuracy between RP and HC across the timepoints of cold challenge, showing global outcomes and seasonal differences

Time	Global			May to July			September to December		
	AUC (95% CI)	Sens	Spec	AUC (95% CI)	Sens	Spec	AUC (95% CI)	Sens	Spec
Basal	0.723 (0.592–0.854)	95.6%	36.4%	0.835 (0.648-1.000)	91.3%	60%	0.826 (0.683–0.968)	86.3%	50%
0 min	0.684 (0.544–0.824)	91.1%	36.4%	0.909 (0.761-1.000)	100%	80%	0.659 (0.475–0.844)	86.3%	25%
2 min	0.773 (0.643–0.904)	95.6%	54.6%	0.983 (0.950-1.000)	91.3%	90%	0.752 (0.562–0.942)	90.9%	33.3%
4 min	0.759 (0.639–0.880)	91.1%	45.5%	0.957 (0.895-1.000)	86.9%	80%	0.750 (0.585–0.915)	77.2%	50%
6 min	0.733 (0.604–0.862)	91.1%	45.5%	0.874 (0.721-1.000)	95.6%	80%	0.723 (0.549–0.898)	86.3%	25%
8 min	0.754 (0.623–0.885)	95.6%	50.0%	0.893 (0.734-1.000)	95.6%	80%	0.754 (0.588–0.920)	90.9%	41.6%
10 min	0.712 (0.580–0.843)	91.1%	36.4%	0.904 (0.768-1.000)	95.6%	80%	0.663 (0.480–0.846)	86.3%	16.6%

When evaluating the period from May to July, ARTIX accuracy was considerably increased, with an AUC always higher than 0.83. Sensitivity was confirmed to be very high, and specificity was greatly increased with percentages of 80% for most of the timepoints. The best timepoint for ARTIX accuracy during the period from May to July was once again 2 min after the cold challenge with AUC 0.983 (95%CI 0.950-1.000), sensitivity 91.3% and specificity 90%.

When evaluating the period from September to December, ARTIX accuracy had a slight decline compared to the global evaluation, although maintaining an AUC almost always greater than 0.7. Sensitivity was slightly reduced ranging from 77.2 to 90.9%, whereas specificity had a considerable decline. The best timepoint for ARTIX accuracy during the period from September to December was at baseline with AUC 0.826 (95%CI 0.683–0.968), sensitivity 86.3% and specificity 50%. See Additional file 1 for ROC curves and confusion matrix for each timepoint of cold challenge with seasonal differences.

## Discussion

The possibility of carrying out frequent assessments represents a key aspect for optimal management of RP. Given the unfeasibility of such frequent live medical visits, patient self-assessment becomes a crucial issue. So far, the Raynaud’s Condition Score and the ASRAP questionnaire have been proposed as reliable patient-reported outcomes for the self-monitoring of RP [5, 6]. However, tools for the objective self-quantification of RP severity are still lacking, and their availability would be a valuable addition to the tools we have for remote monitoring. In this study we developed ARTIX as a novel machine learning-driven method for assessing RP through mobile phone photography. This tool was specifically designed to complement patient-reported severity assessments, offering an objective, image-based quantification of RP burden to enhance both clinical evaluation and patient self-monitoring.

Given their popularity and ease of use, smartphones are an excellent technology for potential home use by patients. For example, the use of smartphone photography has been proposed as a feasible outcome measure in SSc patients for the assessment of DUs [17]. Furthermore, AI is carving out a considerable role in rheumatology, particularly in SSc, being used for a wide range of purposes. In fact, it has been applied to transcriptomics for the classification of SSc patients into intrinsic molecular subsets as well as in histology for the identification of biomarkers for clinical severity and improvement [18, 19]. Machine learning has also been used for diagnostic and prognostic purposes, for example aiding in the early detection of SSc patients with pulmonary arterial hypertension and in the identifications of predictors for a better response to immunosuppressants [20, 21]. An initial attempt to apply AI to hand photographs was proposed by Norimatsu et al. who imaged palm and dorsum of the hands from SSc and non-SSc patients and then used convolutional neural networks to examine the accuracy in distinguishing SSc hands [22]. More recently, Dinsdale et al. proposed a smartphone app to monitor RP during attacks demonstrating good correlations with Raynaud’s Condition Score [23]. The ARTIX score we developed here is not only able to accurately discriminate between RP and HC, but above all provides a quantification of RP burden and it is intended for monitoring even under normal conditions and not only during the colour change that occurs during the RP attack, thus aiding and complementing patient reported-outcomes in a more comprehensive self-assessment of RP burden.

To validate this machine learning algorithm obtaining face and content validity, multiple ground truths were used, hence evaluating ARTIX performances in the comparison between RP and HC across the different timepoints of the cold challenge taking thermography as a reference. In fact, multicentric studies have already widely demonstrated the validity and reliability of mobile thermography for the assessment of the response to cold challenge in RP and SSc patients [11, 12]. In this sense thermography could be considered a gold standard but given the need of specialised thermal sensors it is not feasible for remote monitoring. Furthermore, we sought to evaluate ARTIX outcomes when applied in non-standardized settings, i.e. during different seasons and with different mobile phones. The results obtained with ARTIX are comparable with those of a validated and reliable method such as thermography, but with the advantage of the feasibility and great availability of mobile phones compared to a specialized instrument such as the thermal camera. More in detail, ARTIX paralleled thermography in highlighting significant differences between RP and HC across all the timepoints of the cold challenge. Although both methods are prone to seasonal influence, ARTIX manages to maintain its discriminatory capacity, performing even better than thermography in the first timepoints after the cold challenge carried out in the coldest period from September to December. ARTIX best classification performances were achieved both globally and in the period from May to July two minutes after cold stimulation, which suggests that the main difference between RP and HC lies in the rewarming process. On the other side, the fact that in the period from September to December ARTIX performed its best at baseline is an excellent result in view of a future home implementation. In fact, RP classically shows off more frequently in colder months and the quantification of its severity becomes a crucial issue during this period. Moreover, cold challenge is not something to be reproduced at patient’s home​, hence having a tool that performs well without an “extra” cold stimulation is exactly what is needed for patient self-assessment at home. However, since the diagnosis of RP is necessarily a clinical act, the main goal of ARTIX is not to discriminate between RP and HC but rather to become a feasible tool for the patient self-assessment of RP severity. In this context, some differences emerged in the comparison with thermography, for example the percentage of patients who returned to baseline values at the tenth minute or the aforementioned failure of thermography to detect differences during basal assessments in colder months. These evidence seem to suggest that ARTIX does not represent an estimate of the temperature, but rather expresses a microvascular index of the fingers. Consistent with this hypothesis, the features associated with more severe disease and impaired vascularity such as autoantibodies other than ACA, late NVC pattern and dcSSc showed considerably lower ARTIX scores, whereas treatment with vasoactive drugs was associated with higher values. The lack of significance regarding the history of DUs may find an explanation in the fact that all SSc patients with previous DUs were obviously treated with vasoactive drugs and this may have concealed possible relevant outcomes. Notably, we did not aim to develop this tool for prediction of DUs as there have been other successful efforts in this field [24]. No ARTIX differences were found between primary and secondary RP, consistent with several studies that did not observe any perfusion difference between primary and secondary RP [25–27]. However, all the aforementioned associations with RP-specific characteristics were not among the purposes of the study. We are aware we do not have the statistical power to assess these significances, so all these data have to be considered as exploratory and hypothesis generating. The same applies to the role of ARTIX as a microvascular index for the fingers, future efforts should be directed to investigate which pathophysiological aspect most influences the obtained score (for example peripheral blood perfusion, oxygen saturation or skin tone).

The fairly small cohort is a limitation of this work, along with the lack of data on smoking habits. Unfortunately, the study was not powered to evaluate the possible influence of skin tone on ARTIX: in view of its future implementation, this aspect will need to be explored on a cohort with heterogeneous ethnicity. Moreover, six photographs (out of 469) were not analysable because of blurriness. Another limitation is that the observed seasonal influence may be at least in part driven by the fact that examinations did not take place in a temperature-controlled room, but in a routine hospital clinic room. This could be seen as a methodological limitation, but it increases the real-life value of ARTIX, which is intended for patient self-assessment at home. In this regard, a future implementation of ARTIX with pictures acquired directly form patients is next step in the validation of the tool, and these studies are currently ongoing. Once validated, ARTIX could serve as a tool for measuring RP severity over time as well as for monitoring the response to vasoactive drugs. Larger studies will be also able to assess whether the distribution of ARTIX values in each finger could be useful in assessing primary vs. secondary Raynaud’s or disease progression of secondary RP patients over time from VEDOSS to limited or diffuse cutaneous SSc [3].

## Conclusion

Applying AI to the analysis of mobile phone photographs we developed ARTIX, a novel tool to objectively quantify RP severity. We used an already validated and reliable method for RP assessment such as mobile thermography to check ARTIX performance in the comparison between RP and HC, obtaining excellent congruence between the two methods. Our preliminary results encourage a real-life large-scale study on ARTIX use to complement patient-centered self-assessment of RP severity.

## Electronic supplementary material

Below is the link to the electronic supplementary material.

Supplementary Material 1

## Data Availability

No datasets were generated or analysed during the current study.
