# Supplementary material for: Artificial intelligence-based Raynaud’s quantification index (ARTIX): an objective mobile-based tool for patient-centered assessment of Raynaud’s phenomenon
Source: Arthritis Res Ther. 2025 Jun 3;27:120. doi: 10.1186/s13075-025-03569-w (PMC12131432; doi:10.1186/s13075-025-03569-w)
Supplement: Supplementary file 1 — Supplementary Material 1 [file 13075_2025_3569_MOESM1_ESM.pdf]

Additional file 1. ARTIX classification accuracy with ROC curve and confusion matrix for each timepoint of cold challenge with seasonal differences.

Basal

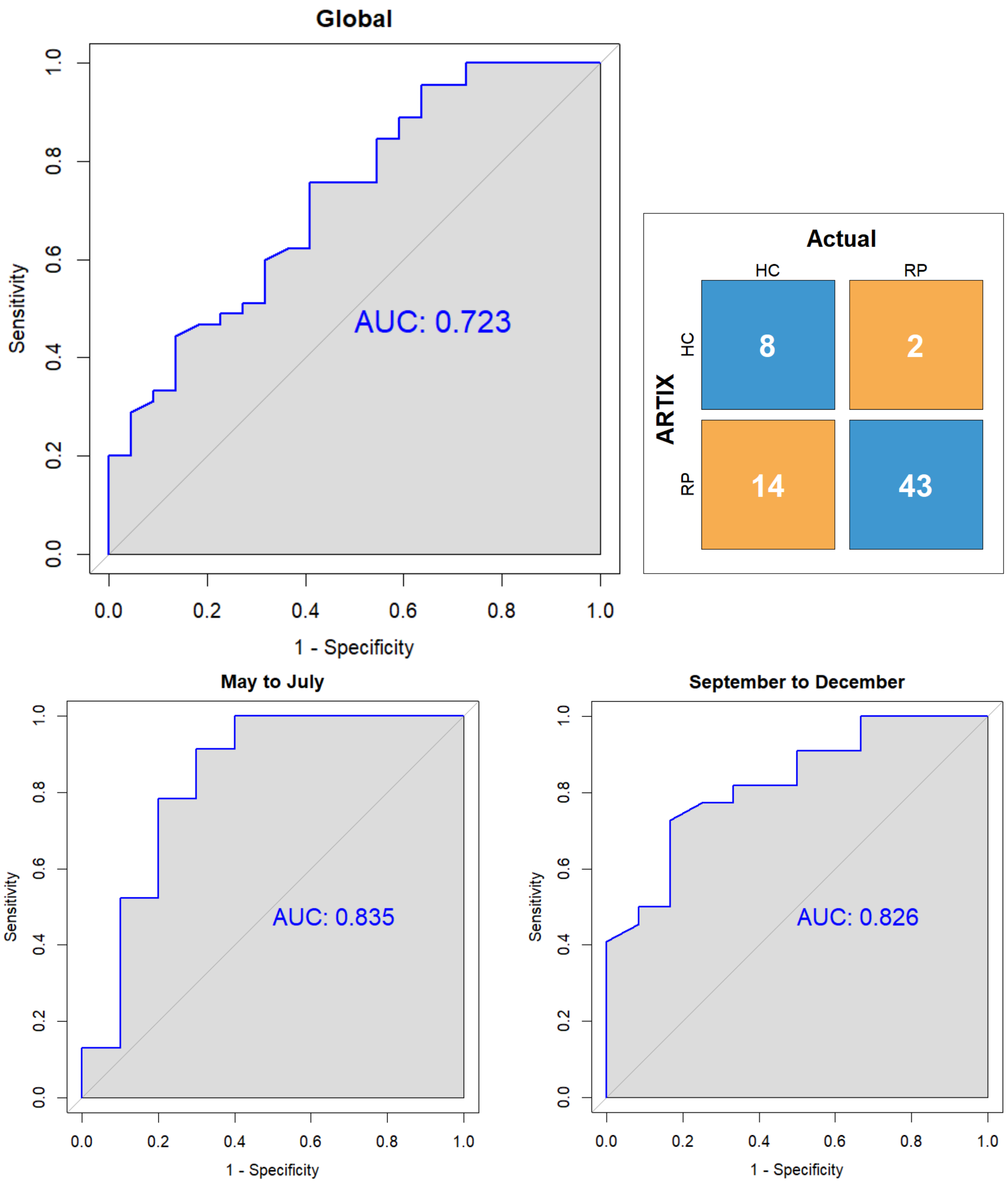

0 min

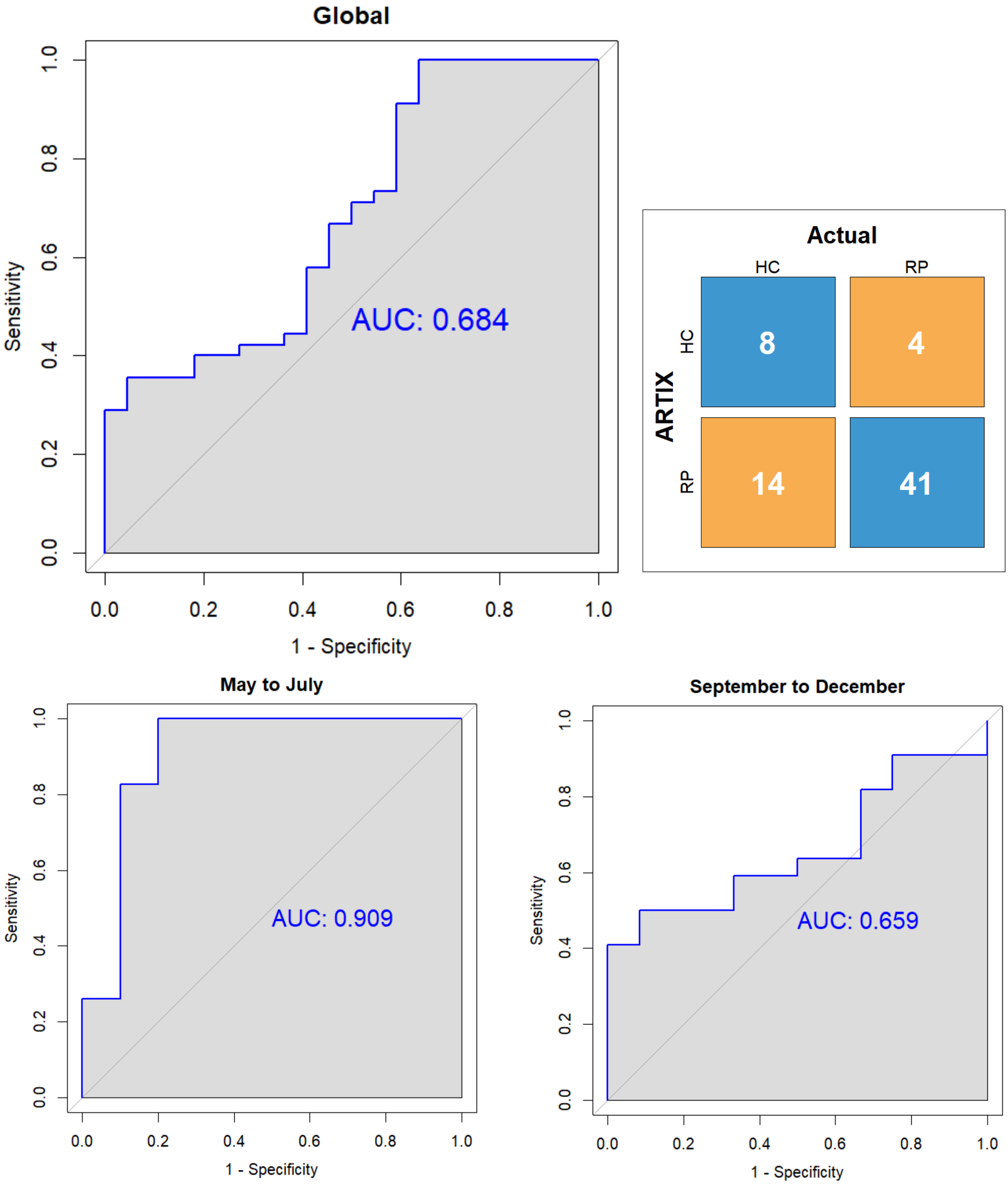

Global
AUC: 0.684
Sensitivity
1 - Specificity
0.0
0.2
0.4
0.6
0.8
1.0
Actual
HC
RP
ARTIX
HC
RP
8
4
14
41
May to July
AUC: 0.909
September to December
AUC: 0.659

2 min

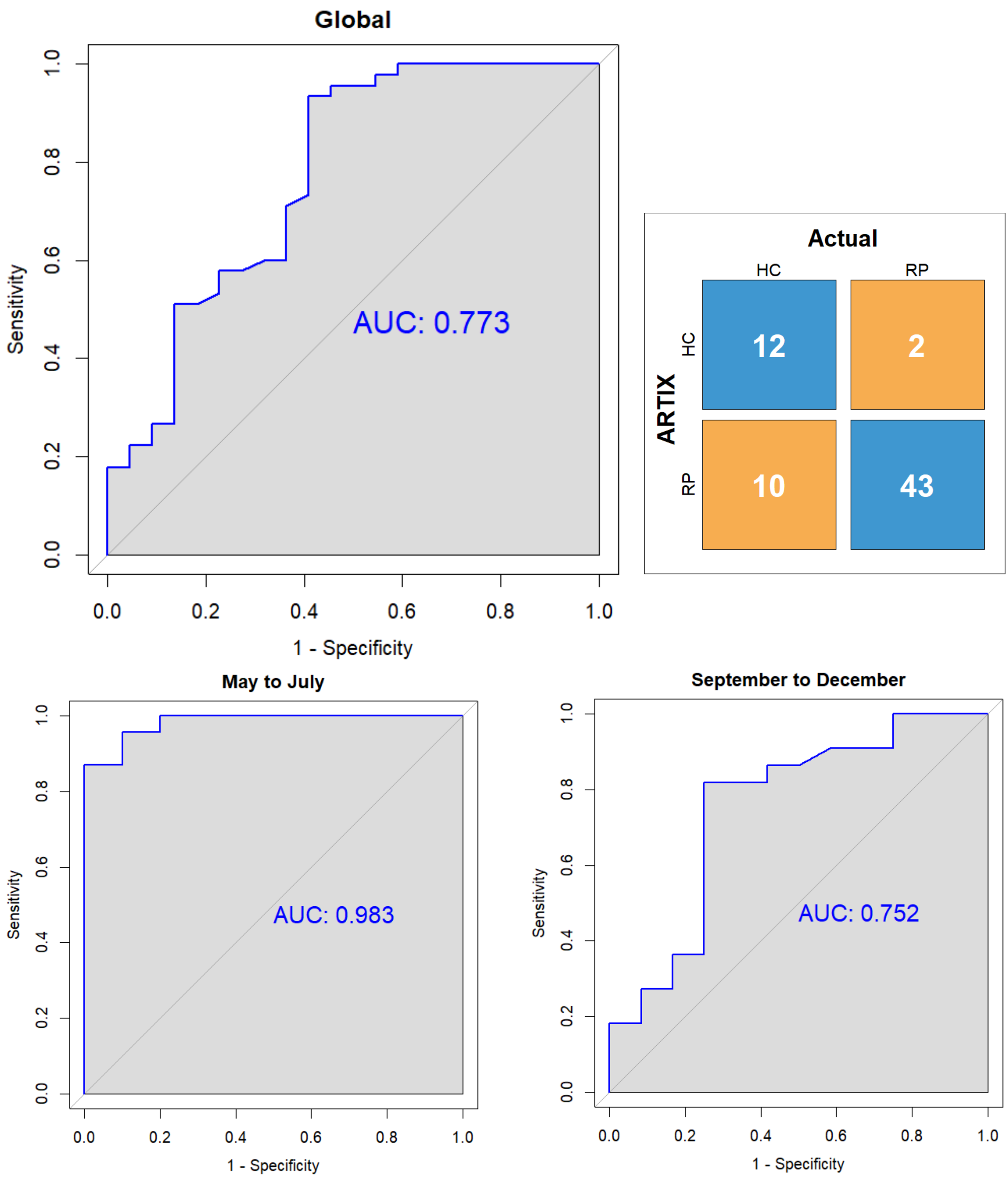
Global
Sensitivity
1 - Specificity
0.0
0.2
0.4
0.6
0.8
1.0
AUC: 0.773
Actual
HC
RP
ARTIX
HC
RP
12
2
10
43
May to July
AUC: 0.983
September to December
AUC: 0.752

4 min

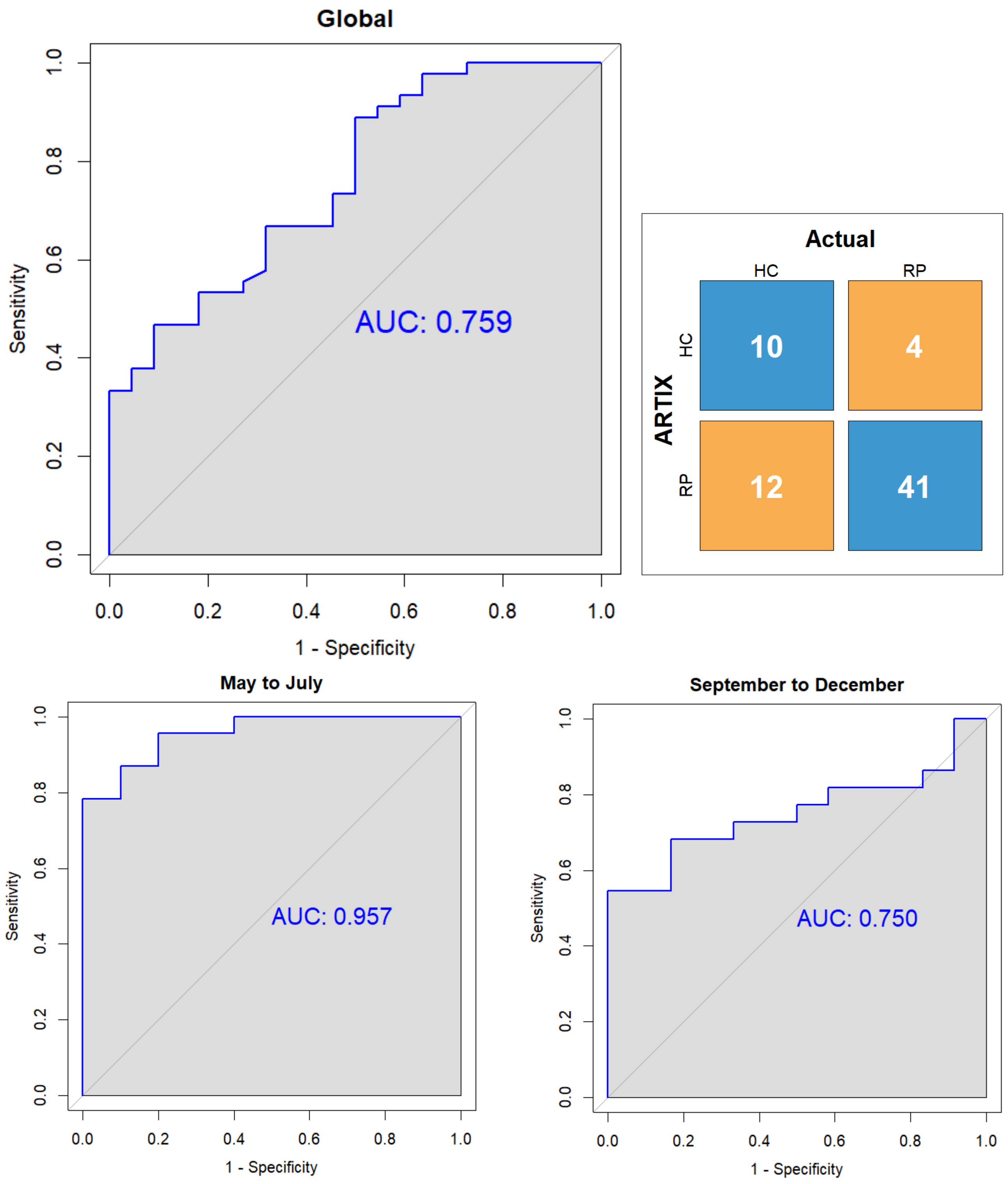
Global
Sensitivity
1 - Specificity
AUC: 0.759
Actual
HC
RP
ARTIX
HC
RP
10
4
12
41
May to July
Sensitivity
1 - Specificity
AUC: 0.957
September to December
Sensitivity
1 - Specificity
AUC: 0.750

6 min

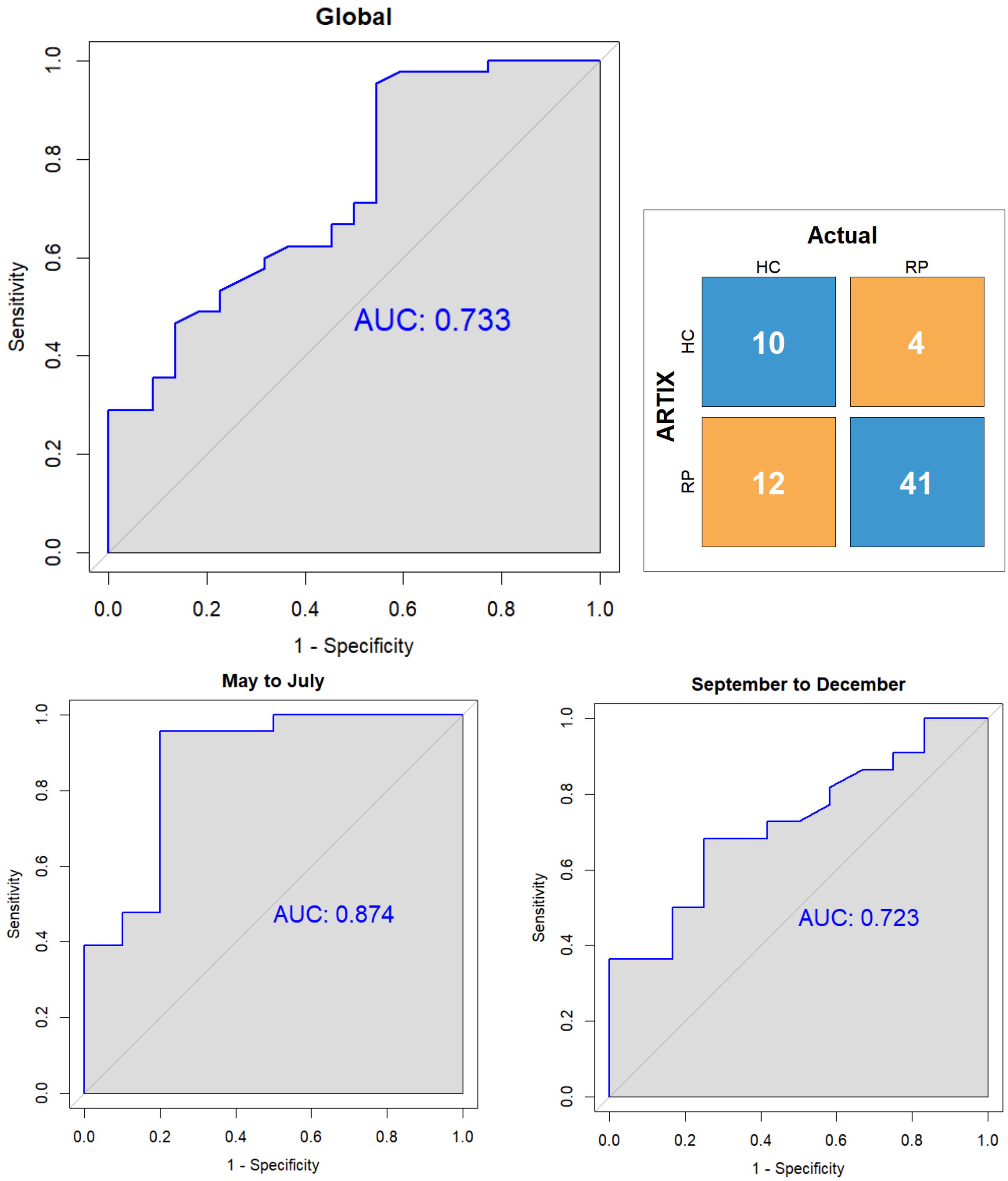
Global
Sensitivity
1 - Specificity
0.0
0.2
0.4
0.6
0.8
1.0
AUC: 0.733
Actual
HC
RP
ARTIX
HC
RP
10
4
12
41
May to July
Sensitivity
1 - Specificity
AUC: 0.874
September to December
Sensitivity
1 - Specificity
AUC: 0.723

8 min

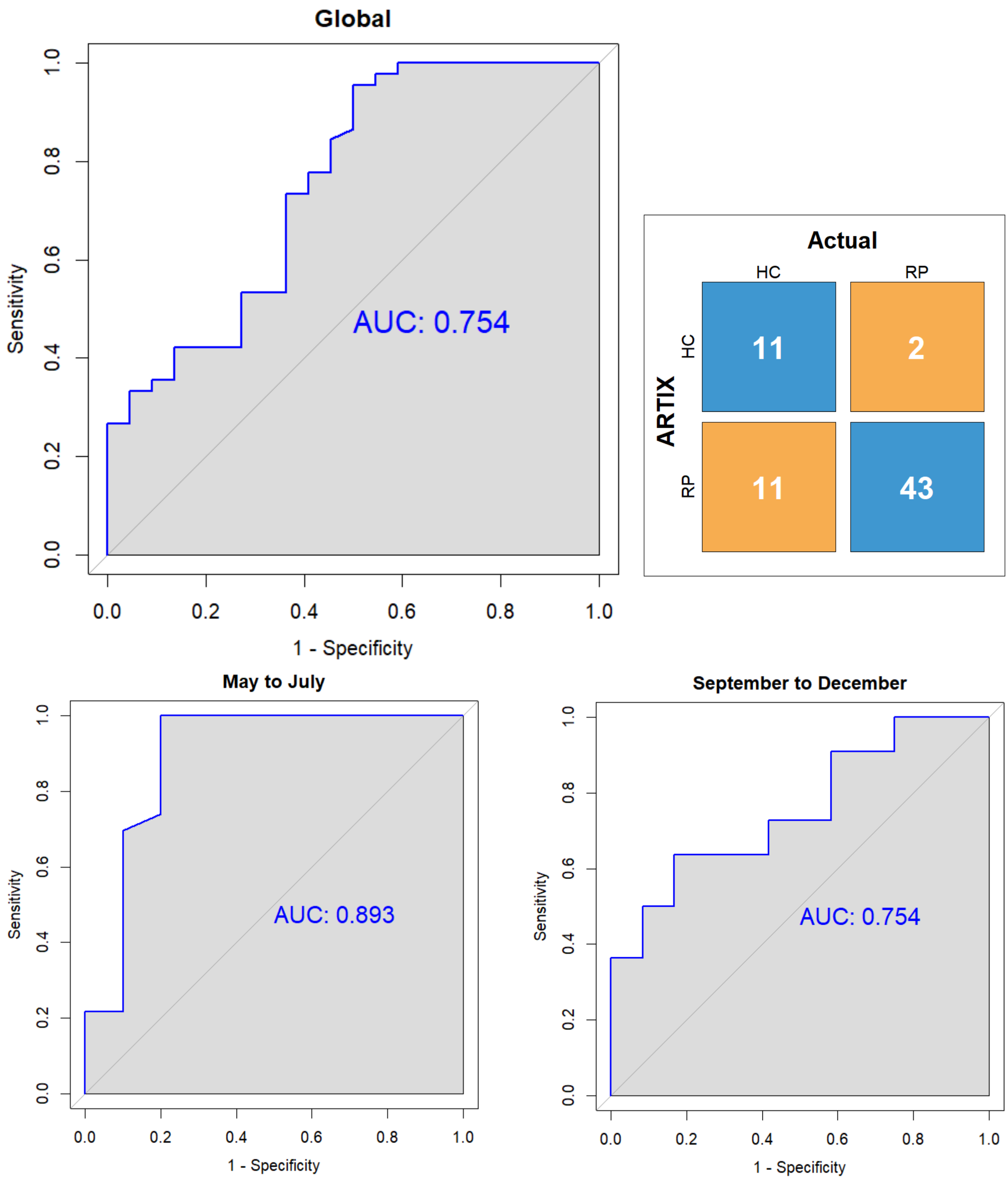
Global
AUC: 0.754
Sensitivity
1 - Specificity
Actual
HC
RP
ARTIX
HC
RP
11
2
11
43
May to July
AUC: 0.893
Sensitivity
1 - Specificity
September to December
AUC: 0.754
Sensitivity
1 - Specificity

10 min

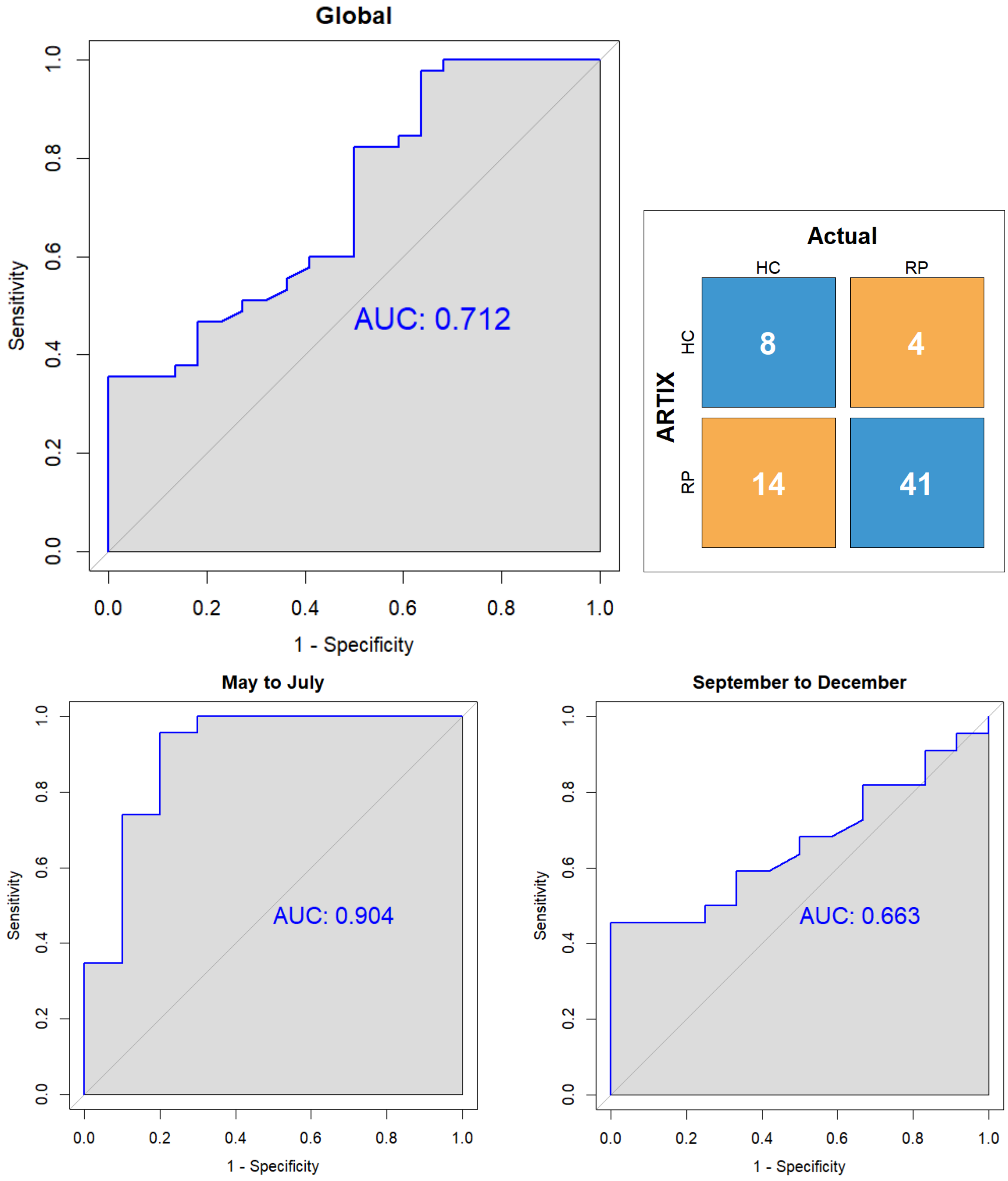
Global
Sensitivity
1 - Specificity
0.0
0.2
0.4
0.6
0.8
1.0
AUC: 0.712
Actual
HC
RP
ARTIX
HC
RP
8
4
14
41
May to July
Sensitivity
1 - Specificity
AUC: 0.904
September to December
Sensitivity
1 - Specificity
AUC: 0.663
